# Effect of Bivalent Vaccines against *Vibrio anguillarum* and *Aeromonas salmonicida* Subspecie *achromogenes* on Health and Survival of Turbot

**DOI:** 10.3390/vaccines9080906

**Published:** 2021-08-14

**Authors:** Yolanda Torres-Corral, Albert Girons, Oscar González-Barreiro, Rafael Seoane, Ana Riaza, Ysabel Santos

**Affiliations:** 1 Departamento de Microbiología y Parasitología, Instituto de Análisis Químico y Biológico (IAQBUS), Universidade de Santiago de Compostela, 15782 Santiago de Compostela, Spain; 2 Ictiovet S.L., 08025 Barcelona, Spain; albert.girons@ictiovet.com; 3 Stolt Sea Farm, Edificio Quercus, 15707 Santiago de Compostela, Spain; o.gonzalez@stolt.com (O.G.-B.); a.riaza@stolt.com (A.R.); 4 Departamento de Microbiología y Parasitología, Facultad de Medicina y Odontología, Universidade de Santiago de Compostela, 15782 Santiago de Compostela, Spain; rafael.seoane@usc.es

**Keywords:** atypical furunculosis, vibriosis, autogenous vaccine, turbot, adjuvants

## Abstract

The efficacy of intraperitoneal injection of an oil-based bivalent autogenous vaccine and the commercial vaccine AlphaJect 3000 (Pharmaq AS) to prevent atypical furunculosis and vibriosis in turbot was analyzed. The effect of both vaccines on health parameters and survival of fish after challenge with *V. anguillarum* and *A. salmonicida* subsp. *achromogenes* was tested. The autogenous vaccine conferred high levels of protection and long-lasting immunity against both pathogens with a single dose. However, severe side effects were observed in turbot injected with this autovaccine and minor negative effects with the AlphaJect 3000 vaccine and the adjuvant Montanide or Eolane. All vaccinated fish showed remarkable antibody agglutination titers, higher than those of control fish, which were maintained 160 d after vaccination. In conclusion, the autogenous bivalent vaccine induces long-lasting protection against atypical furunculosis and vibriosis in turbot, after administration of a single dose, at the cost of high side effects in fish. Therefore, the development of new vaccines should focus on autovaccines and the use of liquid paraffin adjuvants that increase protection with reduced or no side effects.

## 1. Introduction

The culture of turbot, *Scophthalmus maximus* (L.), in aquaculture started in the 1970s in Scotland (UK). Since then, turbot has become one of the main species farmed in Galicia (Spain) and Portugal, in small quantities in other European countries such as France, Norway, Denmark, Holland, and intensively produced in China. Turbot is mainly cultivated in land-based farms with sea water in open flow or using a recirculation aquaculture system (RAS) at temperatures between 12 °C to 20 °C [1]. Several bacterial diseases have been a limiting factor in turbot culture, including infections caused by the devastating fish pathogens *Aeromonas salmonicida* and *Vibrio anguillarum* [2]. Both bacteria are responsible for hemorrhagic diseases with almost identical clinical signs, such as general hemorrhagic septicemia, external lesions, hemorrhage on the fins, and bloody discharges from the vent [2,3].

Furunculosis in fish is a globally distributed bacterial infection caused by *Aeromonas salmonicida.* For years, this disease was thought to have a predilection for salmonids. However, over the years, the apparent host range of the pathogen has expanded considerably, being isolated from a wide variety of diseased fish [2,4]. Four subspecies of *A. salmonicida* have been isolated from fish with furunculosis, such as *A. salmonicida* subsp. *salmonicida*, *A. salmonicida* subsp. *masoucida*, and *A. salmonicida* subsp. *achromogenes* and *A. salmonicida* subsp. *smithia* [5]. *A. salmonicida* subsp. *salmonicida* is the main causative agent furunculosis, also called “typical furunculosis”. The other subspecies are referred to as “atypical strains” which produce “atypical furunculosis” [5,6]. The clinical signs of typical and atypical furunculosis are indistinguishable [2].

Vibriosis in aquaculture is a bacterial disease caused by different species of the genus *Vibrio*, *Listonella*, and *Aliivibrio* [4,6]. The main pathogen causing epizootics in all marine fish species is *Vibrio anguillarum*. This bacterium is widely distributed throughout the world and affects a huge variety of wild and farmed fish species, resulting in high economic losses in the aquaculture sector [2]. Although a total of 23 serotypes of this species have been described, most of the isolates involved in mortalities in Europe, the USA, and Japan belong to the serotypes O1 and O2 (subgroups O2α and O2β) [2,4,6,7].

Traditionally, bacterial threats to aquaculture have been addressed using antimicrobials, including chemotherapeutics, disinfectants, antibiotics, and vaccines. However, the use and misuse of antimicrobials can lead to a rapid increase in the frequency of bacterial resistance, which has a negative impact on the effectiveness of these agents in controlling infectious diseases in aquaculture, the possible transfer of resistance genes from bacteria in the aquatic environment to other bacteria and the possibility of resistance spreading to human pathogens. Careless use of antimicrobials in aquaculture also leads to the possible accumulation of residues in aquaculture products. Consequently, current legislation on the use of antimicrobials in aquaculture has been tightened [8]. The development of infectious disease prevention measures using vaccines is, therefore, a desirable alternative in modern aquaculture. Successful vaccination requires both the development of effective products and their correct use. Numerous vaccine formulations for the control of furunculosis [2,9,10,11] and vibriosis [2,9,12,13,14,15,16] in fish have been developed. In recent years most vaccine development programs have concentrated on multivalent products [10,17,18,19] and adjuvants [20] have become important tools for increasing vaccine potency. In fact, most of the vaccines commercially available are polyvalent oil-adjuvanted vaccines. However, difficulties to achieve effective prophylaxis using these vaccines, coupled with the lack of availability and access to authorized vaccines and the legal restrictions on their use in fish species other than licensed species in the EU, has increased the interest in the development and application of autovaccines in aquaculture systems.

This study aimed to test the efficacy of oil-based commercial and autogenous vaccines against atypical furunculosis and vibriosis in farmed turbot. The impact of these vaccines on health parameters, and fish survival after an experimental challenge was assessed.

## 2. Materials and Methods

The studies presented in this manuscript were approved by the Committee of Bioethics of the Universidade de Santiago de Compostela (USC).

### 2.1. Bacterial Strains

Virulent strains of *Aeromonas salmonicida* subsp. *achromogenes* and *Vibrio anguillarum* originally isolated from diseased turbot during natural epizootics in a Norwegian farm were used for vaccine formulation and challenge tests. The taxonomic position of the strains was confirmed by standard serological and molecular methods [2,21]. Bacteria were grown on tryptic soy agar supplemented with 0.5% NaCl (*w*/*v*) (TSA-1) at 18 °C for 48 h. Stock bacterial cultures were frozen at −80 °C in Microbank^TM^ commercial medium (Pro-Lab Diagnostics, Richmond Hill, ON, Canada).

### 2.2. Vaccines

The paraffin-liquid adjuvanted vaccine AlphaJect 3000 (Pharmaq AS, Overhalla, Norway), designed for salmonid fish, and one autogenous vaccine adjuvanted with non-mineral oil, developed in this study for cultured turbot, were comparatively evaluated. The vaccine AlphaJect 3000 (Pharmaq AS, Overhalla, Norway) has been prepared using *A. salmonicida* subsp. *salmonicida* strain AL 2017 and two strains of *V. anguillarum* of the serotypes O1 (strain AL 112) and O2α (strain AL 104). The concentration of bacteria included in this vaccine is not specified and a paraffin liquid was used as an adjuvant. The strain R0.16.01.01 of *A. salmonicida* subsp. *achromogenes* and the strain of *V. anguillarum* R0.12.04.01 of the serotype O2α, both originally isolated from diseased turbot in Norway were used to prepare the autogenous vaccine. To produce this vaccine, the bacteria were pre-cultured in 100 mL of tryptone soy broth with 1% NaCl (*w*/*v*) (TSB-1) for 48 h. After that time, 10 mL of these cultures were inoculated into a 1 L volume of TSB-1 and incubated for 48 h at 20 °C with shaking at 100 rpm. The cultures were inactivated by the addition of formalin to 0.3% (*v*/*v*) with incubation at room temperature for 2 h and at 4 °C for 24 h. Inactivation was confirmed by the absence of growth of 1 mL volumes in TSB-1 and marine broth (MB) after incubation for 72 h at 20 and 37 °C. To prepare the oil-adjuvanted autogenous bivalent vaccine, pure inactivated cultures of each bacterium were combined and mixed with the non-mineral oil adjuvant Montanide^TM^ (ISA 763A VG; Seppic, France) in a ratio of 25:75 to obtain a stable fluid emulsion with a final concentration of 5 × 10^9^ cells/mL of *V. anguillarum* and 1 × 10^10^ cells/mL of *A. salmonicida* susp *achromogenes*. Sterility was confirmed by spreading the vaccines on TSA-1 and MA plates incubated at 20 and 37 °C for 72 h.

### 2.3. Experimental Fish, Rearing Conditions, and Vaccination Regimes

A total of 1325 certified disease-free turbot with mean weights of 31 ± 2 g were used in the experiments. Sand-filtered seawater was maintained at 16 °C with an oxygen content of more than 8 mg/L and a salinity of 32 ppt was used to maintain the fish. Fish were fed a commercial diet during the experiments.

**Figure 1:**
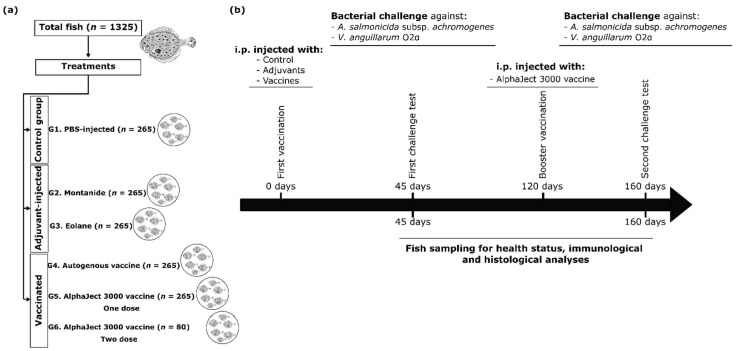
Experimental design (**a**), vaccination strategy, and sampling procedures (**b**).

For the vaccination experiments, the fish were distributed into five equal groups (G1, G2, G3, G4, and G5) of 265 individuals each and placed in equal separate tanks. The experimental design and vaccination strategy are outlined in Figure 1. The control group (G1) consisted of 265 fish injected intraperitoneally (i.p.) with 0.1 mL phosphate-buffered saline (PBS, pH 7.4). Fish in groups 2 (G2) and 3 (G3) were injected i.p. with PBS combined in a 25:75 ratio with the non-mineral oil adjuvant Montanide^TM^ (ISA 763A VG; Seppic, France) or with Eolane 130 liquid paraffin (Total España, Madrid, Spain). Fish in groups G4 and G5 were injected i.p. with 0.1 mL of autogenous and commercial vaccine, respectively. Four months later, a group of 80 fish vaccinated with AlphaJect 3000 (Pharmaq AS, Overhalla, Norway) was retrieved from tank G5, boosted by i.p. injection with the same vaccine and dose, and placed in a new tank (G6). Groups of turbot were marked along the fin margins with a visible implantation fluorescent elastomeric dye. The turbots were kept in the farm facilities until they were transported to the Aquarium facilities of the Faculty of Biology of the University of Santiago de Compostela for analysis. On arrival, the fish were acclimatized in 100 or 300 L tanks at 18 ± 1 °C with a seawater flow rate of 5 mL/min. Prior to any manipulation, fish were anesthetized with methane tricaine sulphonate (MS222, Sigma Aldrich, Madrid, Spain, 60 mg/L). During the experiments, the maintenance conditions (feeding, temperature, water flow, oxygenation) were monitored daily for each group of fish to minimize the tank effect.

### 2.4. Sampling Procedures and Analysis of Fish Health Status

Fish were sampled on the initial day and after 45 d and 160 d of the treatments for health status, immunological, and histological analyses (Figure 1b). Five to twenty fish were examined from each group at each time point. In each sampling, blood samples were collected from the caudal vessels of anesthetized fish using heparinized syringes for hematocrit determination. Hematocrit was determined by centrifugation technique, and the results reported as a percentage. After blood sampling, all fish were examined for the presence of intraperitoneal adhesions according to the Speilberg index (Ss) [22], assigning the value 0 when no visual adhesion and 6 as the highest value where extensive adhesions in the peritoneal cavity are observed. With this aim, the fishes were sacrificed by an overdose of MS 222 (180 mg/L) and a standard necropsy was performed and visual appearance of the abdominal cavity (presence of adherence and/or granulomas) of fish was evaluated. Evaluation of side effects was in all cases carried out by the same person to avoid bias due to inter-observer variation. In addition, samples from the heart, liver, and kidney were taken to evaluate histological alterations after vaccine administration. Tissue samples were fixed in 10% neutral buffered formalin for at least 24 h, embedded in paraffin wax, sectioned at 2–3 μm in thickness, and stained with haematoxylin–eosin (H&E) for light microscopy observation. Histological features of the examined organs were semi-quantitatively evaluated following this criterion: no lesions (0), low (1), moderate (2), and severe (3). Half scores were used for intermediate situations.

At each sampling point, data of corporal weight (W), visceral weight, and hepatic weight were recovered and used to determine the hepatosomatic index (HSI) and viscerosomatic index (VSI), expressed as a percentage. The HSI index was determined using the formula: HSI (%) = 100 × (liver weight [g] /whole fish weight [g]). Similarly, the VSI index was calculated using the formula: VSI (%) = 100 × (viscera weight [g]/whole fish weight [g]).

The respiratory burst activity of the turbot leucocytes was also determined by flow cytometry as previously described [23]. Briefly, the head kidney was aseptically collected and transferred to 5 mL of serum-free culture minimum essential medium (MEM-Earle, Biochrom, Cambridge, UK) supplemented with 100 IU/mL penicillin, 100 μg/mL streptomycin (Biochrom), and 10 IU/mL heparin (Sigma-Aldrich, Madrid, Spain). The cell suspensions were obtained by forcing the tissues through a nylon mesh (mesh size 100 μm) in cold serum-free MEM. The resulting cell suspensions were placed onto Ficoll-Paque^TM^ PLUS (Amersham Biosciences, Uppsala, Sweden) and centrifuged at 400× *g* for 20 min at 4 °C to remove erythrocytes and residues. The leucocyte-rich interphase was collected with a Pasteur pipette, washed twice, counted using the trypan blue exclusion test of cell viability, and adjusted to 10^6^ viable cells/mL in MEM supplemented with antibiotics and 5% fetal calf serum (MEM-5) (Biochrom). The respiratory activity of leucocytes was quantified as a measure of intracellular reactive species using dihydrorhodamine 123 (DHR, 10 mM, stabilized in DMSO) (Sigma) as an oxidative probe. For measurement of radical production, cells were treated with phorbol 12-myristate-13 acetate (PMA, 0.2 µg /mL in dimethyl sulfoxide) (Sigma-Aldrich, Madrid, Spain) or left untreated as controls. Propidium iodine (PI, 0.1 µg/mL) (Sigma-Aldrich, Madrid, Spain) was added to all samples to gate out PI^+^ cells. The level of intracellular fluorescence was measured in non-stimulated, and PMA stimulated cells. A total number of 1 × 10^4^ events in the cell gate were analyzed. The results were expressed as a stimulation index (SI) which is calculated by dividing the geometric mean fluorescence intensity (GMFI) of treated (PMA) and untreated (Controls) samples. The assays were carried out in triplicate.

Anti-*A. salmonicida* subsp. *achromogenes* and *V. anguillarum* antibody titers were determined by standard microtiter agglutination methods. Briefly, the sera were first diluted 1:2 with PBS and then serially diluted ten-fold with the same solution in a 96-well U-shaped microtiter plate with thorough mixing. Then, 10 µL of bacterial suspensions (10^9^ CFU/mL; absorbance 620 = 220) of *V. anguillarum* (serotype O2α strain RO15.11.2) or *A. salmonicida* subsp. *achromogenes* (strain RO16.02.01.02) were added to each well and the plate was incubated at 20 °C for 18 h. Agglutination titers were estimated as the reciprocal of the highest dilution showing agglutination. An auto-agglutination control was performed by mixing bacterial suspensions and sterile PBS instead of antiserum.

All results were subjected to analysis of variance (ANOVA) with the post hoc Tukey HSD and Bonferroni multiple comparison results with a significance level of *p* < 0.05.

### 2.5. Efficacy of Vaccination

Vaccine efficacy and duration of immunity were determined by an infectious challenge test and determination of the relative survival percentage (RPS) with a control mortality of 60, according to the recommendations of the European Pharmacopoeia [13]. For the challenge test, fish were infected by i.p. injection with virulent strains of *V. anguillarum* RO15.11.2 and *A. salmonicida* subsp. *achromogenes* RO16.02.01.02 after 45 and 160 d of treatments. Forty-five days after vaccination, turbot in each treatment group were challenged with a suspension of *V. anguillarum* strain RO15.11.2 (50 fish from each group) or *A. salmonicida* subsp. *achromogenes* strain RO16.02.01.02 (50 fish from each group). To determine the duration of vaccine-induced immunity, experimental infection with *V. anguillarum* strain RO15.11.2 (30 fish from each group) or with *A. salmonicida* subsp. *achromogenes* strain RO16.02.01.02 (30 fish from each group) was carried out 160 d after vaccination. In addition, fish boosted with AlphaJect 3000 vaccine (Pharmaq AS, Overhalla, Norway), were infected with *V. anguillarum* (30 fish) and *A. salmonicida* subsp. *achromogenes* (30 fish) 40 d after administration of the second dose of vaccine. The number of viable bacterial cells present in the inoculum was determined by seeding 0.1 mL serial dilutions of the bacterial suspensions on TSA-1 plates and counting the colony-forming units (CFU) produced. Challenged fish were maintained at 18 °C ± 1 °C and mortalities were recorded daily until at least 60 percent of specific mortality was reached in the control group. The specificity of mortalities was confirmed by seeding samples from internal organs (kidney and spleen) of dead or moribund fish on TSA-1 plates and recovery of inoculated strains in pure culture.

The efficacy of vaccination was evaluated by plotting for both, vaccinated and control group, specific mortality curves against time from challenge. The time corresponding to 60 percent specific mortality in controls was determined by interpolation and the mortality of vaccinated fish (M) was determined at the time corresponding to 60 percent mortality in controls. The RPS was calculated using the following expression [13]: RPS = (1 − M/60) × 100. The Chi-square test (χ^2^) was also used as a statistical analysis to verify significant differences in survival between vaccinated and control groups after challenge, using a significance level of *p* < 0.05.

## 3. Results

### 3.1. Macroscopic and Histological Effect of Vaccination

Side effects of the immunization of turbot with the autogenous and commercial oil-adjuvanted vaccines and adjuvants were evaluated by visual and histological analysis (Table 1). The highest degree of damage in the peritoneal cavity, significantly higher than the control group (*p* < 0.005), was recorded at 45 d post-vaccination of turbot with the autogenous vaccine (Ss = 5.65 ± 0.75) and the commercial vaccine (Ss = 4.45 ± 1.05) or injection of the adjuvant Montanide^TM^ (Ss = 2.60 ± 0.99). Mild or minor adhesions were detected in fish injected with the adjuvant Eolane (Sc = 0.68 ± 0.95). Similar results were observed after 160 d in fish vaccinated with the autogenous vaccine (Ss = 5.50 ± 0.53) (Table 1), while the lowest Ss values were observed in fish vaccinated with one (Ss = 2.50 ± 1.07) or two doses (Ss = 3.88 ± 0.35) of AlphaJect 3000 vaccine or fish injected with the mixture PBS:adjuvants (Ss = 0.40 ± 0.97).

**Figure 2:**
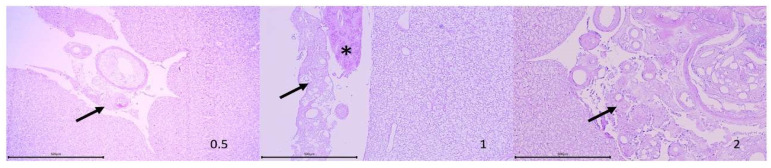
High-power photographs (40×) of liver sections showing a variable incidence of perivisceral granulomatous reaction (arrows). Acinar cells of the exocrine pancreas are indicated with an asterisk (*) and the histological score corresponding to this specific feature is indicated in the lower right corner of each section. Staining: haematoxylin and eosin. Scale bar 100 μM.

Liver samples showed the most significant histological signs. Histological examination of the liver samples from most groups showed an inflammatory granulomatous reaction associated with the hepatic serosa (Figure 2) consisting of a thick fibrous capsule and empty core granulomas, as well as loose fibrous tissue and a variable amount of mixed inflammatory infiltrate. Only fish injected with PBS showed no granulomatous reaction in this region. Fish injected with the adjuvants (Eolane and Montanide), commercial vaccine, and autovaccine showed focal or multiple granulomatous structures (lesion intensity ranging from 0.13 ± 0.23 to 0.94 ± 0.56), associated with the hepatic serosa and occasionally associated with the exocrine pancreas. In these cases, no lesions or alterations were detected in the liver parenchyma in contact with liver serosa or exocrine pancreas tissue.

Intracytoplasmic vacuolation of hepatocytes was also observed, with lesions of varying intensity (values ranging from 1.75 ± 0.53 to 2.0 ± 0.65). These signs are more likely to be associated with differences in food intake than with vaccine injection factors.

Minor, non-specific background histopathological signs were detected in liver parenchyma, kidney, and heart samples, but no other signs were detected that could be clearly related to vaccine or adjuvant injection

### 3.2. Evaluation of Physiological Parameters

The results of the health parameters evaluated are presented in Table 2. After 45 d post-treatment, the mean body weight of fish ranged from 71.0 ± 14.0 g to 88.0 ± 16.0 g for vaccinated turbot and from 94.0 ± 16.0 g to 106.0 ± 17.0 g for fish injected with adjuvants. No significant differences were observed for this parameter with respect to the values found for control fish (94.0 ± 17.0 g) (Table 2). The VSI of turbot vaccinated with autogenous (10.7 ± 1.8%) and commercial (7.6 ± 0.9%) vaccines were significantly higher than that of fish injected with Montanide (6.4 ± 0.8%), Eolane (6.4 ± 0.9%) or PBS (6.0 ± 1.1%) (Table 2). Turbot vaccinated with the autogenous vaccine showed significantly lower HSI (0.7 ± 1.1%) (*p* < 0.05) than fish injected with the commercial vaccine (2.2 ± 0.3%), Eolane (2.0 ± 0.4%), Montanide (2.0 ± 0.3%), or PBS (1.8 ± 0.3%) (Table 2). After 160 d post-immunization, no differences were observed between treatment groups for HSI. The bodyweight of turbot vaccinated with the autogenous vaccine was significantly smaller than that of fish injected with Eolane 130 (339.5 ± 73.0 g) or PBS (304.8 ± 55.0 g) (Table 2). Differences (*p* < 0.05) were also observed between treatments regarding VSI, which was significantly higher in fish vaccinated with the autogenous vaccine (Table 2).

After 45- and 160-d post-treatment, respiratory burst activity (SI) of kidney leukocytes and hematocrit values of turbot vaccinated (groups G4, G5, and G6) or injected with adjuvants (groups G2 and G3) showed no significant differences with respect to those obtained with the control group (G1) (Table 2).

The specific immune response in turbot immunized with autogenous and AlphaJect 3000 vaccines was assessed by measuring serum agglutinating antibody titers at 45- and 160-d post-vaccination, using inactivated whole bacterial cells of *A. salmonicida* subsp *achromogenes* and *V. anguillarum* as antigens. Only sera from fish immunized with the autogenous and the commercial vaccines displayed specific agglutination with the antigens of both pathogens (Table 3). Significative (*p* < 0.05) higher values of agglutinating antibody titer against *A. salmonicida* subsp *salmonicida* were only recorded after 45 d of immunization of fish with the autogenous vaccine. Similar titer values against *V. anguillarum* antigens were observed in fish immunized with the autogenous and commercial vaccines 45 d post-vaccination. After 160 d post-vaccination, only fish vaccinated with a single dose of the autogenous vaccine or two doses of the commercial vaccine AlphaJect 3000 maintained notable antibodies titers against *Aeromonas* and *Vibrio* antigens.

### 3.3. Efficacy of Vaccines

The results of the infective challenge 45 d post-vaccination showed that turbot i.p vaccinated with the oil-based autogenous (group G4) and AlphaJect 3000 (G5 and G6) vaccines were fully protected (RPS = 100; Table 4) following i.p. challenge with *V. anguillarum*. However, only the autogenous vaccine (G4) conferred high levels of protection of turbot against *A. salmonicida* subsp. *achromogenes* (RPS = 83%). One-hundred- and sixty-days post-vaccination, moderate protection against *V. anguillarum* was obtained for both fish immunized with one (G5) or two (G6) doses of AlphaJect 3000 vaccine (RPS = 52 to 60%), and fish immunized with a single dose of the autogenous (G4) vaccine (RPS = 67%). In contrast, increased levels of protection after challenge with *A. salmonicida* subsp. *achromogenes* were observed in turbot vaccinated with one (G5, RPS = 77%) or two doses of AlphaJect 3000 (G6, RPS = 100%) or a single dose of the autogenous vaccines (G4, RPS = 100%). χ^2^ statistical analyses showed significant differences (*p* < 0.05) between the final mortalities of the vaccinated groups (challenged after 45- and 160-d post-immunization) and the respective control groups.

The specific antibody response elicited by the different vaccines correlates with the degree of protection offered by each of them. However, vaccination had no significant effect on the respiratory burst activity of kidney leucocytes or on the hematocrit levels of fish blood.

The influence of the i.p. injection of adjuvants Montanide^TM^ ISA 763A VG and Eolane 130 on survival of fish after challenge with *V. anguillarum* and *A. salmonicida* subsp. *achromogenes* was also evaluated (Table 4). Fish injected with Montanide^TM^ (ISA 763A VG) showed low protection values after challenge with *V. anguillarum* (G2, RPS = 38%). Similar results were obtained for fish injected with the adjuvant Eolane 130 (G3) (Table 4). Statistical analysis showed significant differences (*p* < 0.05) between the final cumulative mortalities achieved in the groups injected with adjuvants (G2 and G3) and the control groups injected with PBS (G1) after 45 d post-vaccination. In contrast, fish injected with both adjuvants showed high protection levels against *A. salmonicida* subsp. *achromogenes*. In most cases, these protection levels (RPS) decrease after 160 d of the injection of the adjuvants (RPS values ranging from 0% to 55% for *A. salmonicida* subp. *achromogenes* and 5% to 7% for *V. anguillarum*; Table 4).

## 4. Discussion

Vibriosis and atypical furunculosis are two of the bacterial diseases causing mortalities in intensively farmed turbot. Although several commercial vaccines exist to prevent vibriosis and furunculosis in fish, most of these vaccines are tested on salmonids and/or contain antigens against *A. salmonicida* subsp. *salmonicida*, which is responsible for typical furunculosis. Therefore, this study aimed to test the efficacy of oil-based commercial and autogenous vaccines against atypical furunculosis and vibriosis in farmed turbot. The impact of these vaccines on health parameters, and fish survival after the experimental challenge was assessed.

Vaccine efficacy can be improved by using adjuvants together with the selected antigens. Among adjuvanted vaccines, formulations with mineral oil adjuvants have been associated with side effects, such as growth impairment [24,25,26], pigmentation, inflammation, and granulomatous lesion formation [25,27,28] and fibrous adhesions in internal organs [26,28,29,30]. In the present study, severe side effects (inflammation, granulomas, and organ adhesion) were observed with turbot immunized with the autogenous vaccines, and lower effects with the commercial vaccine and the adjuvant Montanide. These alterations could be caused by the deposition of adjuvants on or between organs, which infiltrate, forming a vaccine cell mass that facilitates the formation of adhesions [31,32,33]. One hundred- and sixty-days post-treatment, significantly elevated Ss values were still observed in fish immunized with the autogenous and commercial vaccine (one and two doses), but not in fish treated with the adjuvant Montanide^TM^ (ISA 763A VG; Seppic, France) alone. These results seem to indicate that these side effects are caused by interactions of the antigens and the oil emulsion adjuvant [24,34,35]. In contrast, fish injected with the adjuvant Eolane showed only mild or minor adhesions. These results suggest that Eolane is a well-tolerated oily adjuvant for turbot that could be considered in future vaccine development.

Body indexes help to determine the health status of fish by providing specific information related to the function of the selected organ [36]. Among the growth performance parameters analyzed, differences were found for fish weight and HSI, significantly reduced in fish vaccinated with the Montanide-adjuvanted autogenous vaccine. There are divergent opinions on the role of oily adjuvants for observed growth reduction in vaccinated fish. While some authors [37] consider that interacting antigen–adjuvant rather than the adjuvant components themselves are responsible for reduced growth in vaccinated fish, others have postulated that adhesions and lesions caused by vaccination lead to a reduction in feed uptake and growth [38,39].

Microscopically, liver lesions were observed in fish injected with oil-based adjuvants and adjuvanted vaccines, predominantly mild to moderate necrosis and granulomatous structures attached to the serosa and, occasionally, to the exocrine pancreas. Histological alterations such as local necrosis or granulomatous lesions in hepatic tissue are consistent with previous observations in turbot and cod injected with oil-adjuvanted vaccines [31,35]. Intracytoplasmic vacuolation of hepatocytes and non-specific background histopathological signs were detected in the liver parenchyma, kidney, and heart samples. However, these alterations can be commonly observed in healthy fish and should not be considered as a relevant alteration associated with the treatments applied to the fish in the present study.

The hematocrit values have been considered as a simple and non-specific indicator of fish health over the years [40,41,42,43], moreover, this indicator has resulted in an informative index in cultured fish [44]. In the current study, no significant differences were observed between the hematocrit value in the treated fish groups. The respiratory activity of macrophages has also been evaluated because the cellular response plays an important role in host defense mechanisms, demonstrated by their action in coagulation and inflammation processes, and phagocytic activity in infectious processes [45]. However, in this study, no significant differences were found between the stimulation index between vaccinated and control fish after 45 and 160-d post-vaccination.

The efficacy and duration of immunity of the vaccines were tested by an infectious challenge test with *V. anguillarum* and *A. salmonicida* subsp. *achromogenes* and determination of RPS. After 45 d post-treatment, the commercial and autogenous vaccines induced complete protection against *V. anguillarum* (RPS = 100%). However, only the bivalent autogenous vaccine, which included antigens of a strain of *A. salmonicida* subsp *achromogenes* isolated from turbot, conferred high levels of protection (RPS = 83%) against this pathogen after administration of a single dose. A likely explanation for the high levels of protection conferred by the autogenous vaccine against both pathogens could be the use of specific antigens in the appropriate dose, which are important in the stimulation of the immune system of this fish species. Similar results were found comparing vaccines against typical and atypical furunculosis [11,46]. The duration of immunization of the vaccines was tested by infectious challenge test after 160 d post-treatment. Only fish vaccinated with a single dose of autogenous vaccine retained high levels of protection against *V. anguillarum* and *A. salmonicida* subsp. *achromogenes*. The possibility of using bivalent vaccines in a single dose would reduce stress to the fish and reduce costs for the farmers. Therefore, the high levels of protection observed using the autogenous vaccine could represent a significant improvement for both fish health and the farming economy.

## 5. Conclusions

The oil-based autogenous bivalent vaccine developed in this study, conferred high levels of long-term protection against atypical furunculosis and vibriosis after administration of a single dose in turbot, at the cost of high side effects in the fish. Therefore, further studies should be focused on autovaccines and the evaluation of other adjuvants that improve protection with reduced or no side effects. In this sense, the liquid paraffin Eolane 130 evaluated in this study, which is a well-tolerated adjuvant for turbot, is a promising candidate that could be considered in future vaccine development.

## Figures and Tables

**Table 1:** Macroscopic and microscopic effects of fish after treatments. Results are presented as the mean ± standard deviation.

	Macroscopic Effects Spielberg Scale (Ss)		Microscopic Effects Semiquantitative Scale ^a^				
Fish Group	Days Post-Treatment		Liver				
	45	160	Vacuolization	Adhesion on Serosa	Bile duct Fibriosis	Degeneration/Necrosis	Renal tubules Vacuolation
G1	0.00 ± 0.00	0.00 ± 0.00	2.00 ± 0.38	0.00 ± 0.00	1.00 ± 0.00	1.13 ± 0.23	0.43 ± 0.19
G2	2.60 ± 0.99	0.40 ± 0.97	1.94 ± 0.73	0.13 ± 0.23	1.13 ± 0.59	0.44 ± 0.42	0.69 ± 0.37
G3	0.68 ± 0.95	0.40 ± 0.97	1.75 ± 0.53	0.19 ± 0.37	1.00 ± 0.00	0.19 ± 0.37	0.69 ± 0.59
G4	5.65 ± 0.75	5.50 ± 0.53	1.81 ± 0.59	0.88 ± 0.79	1.00 ± 0.00	0.81 ± 0.59	0.88 ± 0.52
G5	4.45 ± 1.05	2.50 ± 1.07	2.00 ± 0.65	0.94 ± 0.56	1.13 ± 0.59	0.69 ± 0.59	1.25 ± 0.93
G6	ND	3.88 ± 0.35	1.81 ± 0.70	0.56 ± 0.56	1.19 ± 0.37	0.94 ± 0.68	0.75 ± 0.46

G1, control group; G2, fish injected with Montanide; G3, fish injected with Eolane; G4, fish immunized with the autogenous vaccine; G5 and G6, fish immunized with one or two doses of AlphaJect 3000 vaccine, respectively. ^a^ Semi-quantitative scale: no lesions (0), low (1), moderate (2), and severe (3). Half scores were used for intermediate situations.

**Table 2:** Health parameters of fish after treatments.

Fish Group	45 D after Treatment					160 D after Treatment				
	Fish Weight (g)	VSI (%)	HSI (%)	Hematocrit	SI	Fish Weight (g)	VSI (%)	HSI (%)	Hematocrit	SI
G1	94.4 ± 17.0	6.0 ± 1.1 ^b^	1.8 ± 0.3 ^b^	36.6 ± 13.0	2.7 ± 1.5	304.8 ± 55.0 ^b^	5.3 ± 0.2 ^b^	1.8 ± 0.4	26.8 ± 2.3	1.7 ± 0.1
G2	94.0 ± 16.0	6.4 ± 08 ^b^	2.0 ± 0.3 ^b^	32.2 ± 11.2	1.0 ± 0.4	285.0 ± 81.0	5.7 ± 0.5 ^b^	1.6 ± 0.3	28.8 ± 4.9	2.0 ± 0.2
G3	105.7 ± 17.0	6.4 ± 0.9 ^b^	2.0 ± 0.4 ^b^	42.3 ± 16.2	ND	339.5 ± 73.0 ^b^	5.6 ± 0.5 ^b^	1.9 ± 0.4	27.2 ± 3.1	1.8 ± 0.3
G4	70.6 ± 14.0	10.7 ± 1.8 ^a^	0.7 ± 1.1 ^a^	36.4 ± 12.6	2.7 ± 0.6	205.2 ± 58.0 ^a^	6.8 ± 0.9 ^a^	1.7 ± 0.2	32.2 ± 6.2	2.4 ± 0.6
G5	88.3 ± 16.0	7.6 ± 0.9 ^a^	2.2 ± 0.3 ^b^	41.0 ± 6.3	2.1 ± 0.5	300.6 ± 42.0	5.6 ± 0.3 ^b^	1.8 ± 0.2	32.6 ± 10.5	2.8 ± 0.3
G6	ND	ND	ND	ND	ND	226.7 ± 46.0	5.7 ± 0.5 ^b^	1.6 ± 0.9	32.3 ± 5.8	2.2 ± 0.1

G1, control group; G2, fish injected with Montanide; G3, fish injected with Eolane; G4, fish immunized with the autogenous vaccine; G5 and G6, fish immunized with one or two doses of AlphaJect 3000 vaccine, respectively. VSI, viscerosomatic index = [Visceral weight (g)/Corporal weight (g)] × 100; HSI, hepatosomatic index = [Liver weight (g)/Corporal weight (g)] × 100; Hematocrit was determined by centrifugation technique, and the results reported as a percentage. SI: Stimulation index = Geometric mean fluorescence intensity of stimulated leukocytes/Geometric mean fluorescence intensity of unstimulated leukocytes. Data with a different superscript (^a, b^) in a column were significantly different ANOVA (*p* < 0.05).

**Table 3:** Results of the agglutination assay using the serum of turbot injected with PBS, eolane, montanide, autogenous (one dose), and commercial vaccines (one and two doses).

Antigens	Fish Groups										
	G1		G2		G3		G4		G5		G6
	45d	160d	45d	160d	45d	160d	45d	160d	45d	160d	160d
*A. salmonicida* subsp. *achromogenes*	<2 ^b^	<2 ^b^	<2 ^b^	<2 ^b^	<2 ^b^	<2 ^b^	1468 ± 878 ^a^	200 ± 0 ^b^	174 ± 68 ^b^	12 ± 10 ^b^	200 ± 0 ^b^
*V. anguillarum* O2α	2 ± 0 ^b^	<2 ^b^	<2 ^b^	<2 ^b^	<2 ^b^	<2 ^b^	637 ± 934 ^b^	140 ± 104 ^b^	406 ± 708 ^b^	16 ± 9 ^b^	650 ± 900 ^b^

G1, control group; G2, fish injected with Montanide; G3, fish injected with Eolane; G4, fish immunized with the autogenous vaccine; G5 and G6, fish immunized with one or two doses of AlphaJect 3000 vaccine, respectively. 45 d and 160 d, 45 and 160 d post-treatment; Results expressed as mean ± standard deviation of agglutination titters using samples of 6 fish per treatment. Data with a different superscript (^a, b^) in a column were significantly different ANOVA (*p* < 0.05).

**Table 4:** Efficacy bivalent vaccines and vaccination procedures carried out in turbot under laboratory conditions.

Fish Group	Vaccination Procedure		Infectious Dose Cells/Fish	%Mortality	RPS_60_
	Primary Dose	Booster Dose			
**Protection against *V. anguillarum* 45 d Postvaccination**					
G1	i.p. injection		1 × 10^9^	60 ^a^	
G2	i.p. injection			37 ^b^	38
G3	i.p. injection			35 ^b^	42
G4	i.p. injection			0 ^b^	100
G5	i.p. injection			0 ^b^	100
**Protection against *A. salmonicida* subsp. *achromogenes* 45 d postvaccination**					
G1	i.p. injection		9.9 × 10^8^	60 ^a^	
G2	i.p. injection			4 ^b^	93
G3	i.p. injection			4 ^b^	93
G4	i.p. injection			10 ^b^	83
G5	i.p. injection			30 ^b^	50
**Protection against *V. anguillarum* 160 d postvaccination**					
G1	i.p. injection		6.6 × 10^10^	60 ^a^	
G2	i.p. injection			57	5
G3	i.p. injection			56	7
G4	i.p. injection			29 ^b^	52
G5	i.p. injection			20 ^b^	67
G6	i.p. injection	i.p. injection		24 ^b^	60
**Protection against *A. salmonicida* subsp. *achromogenes* 160 d postvaccination**					
G1	i.p. injection		1.4 × 10^10^	60 ^a^	
G2	i.p. injection			78	0
G3	i.p. injection			27 ^b^	55
G4	i.p. injection			0 ^b^	100
G5	i.p. injection			14 ^b^	77
G6	i.p. injection	i.p. injection		0 ^b^	100

G1, control group; G2, fish injected with Montanide; G3, fish injected with Eolane; G4, fish immunized with autogenous vaccines; G5 and G6, fish immunized with one or two doses of AlphaJect 3000 vaccine, respectively. RPS60, Relative survival rate 60% = (1-M/60) × 100 (European Pharmacopoeia 7.0, 2010). Calculation based on the percentage of specific mortality in the vaccinated group (M), obtained by interpolation at the time when 60 percent of the specific mortality in the control group is reached. Data with a different superscript (^a, b^) in a column were significantly different χ^2^ (*p* < 0.05).

## Data Availability

The dataset used and/or analyzed during the current study are available from the corresponding author on reasonable request.
